# Progress in the Metabolomics of Acute Coronary Syndrome

**DOI:** 10.31083/j.rcm2407204

**Published:** 2023-07-14

**Authors:** Fu Zhang, Bo Li, Hongling Su, Zhaoxia Guo, Hai Zhu, Aqian Wang, Kaiyu Jiang, Yunshan Cao

**Affiliations:** ^1^Department of Cardiology, Pulmonary Vascular Disease Center (PVDC), Gansu Provincial Hospital, 730000 Lanzhou, Gansu, China; ^2^The First Clinical Medical College of Gansu University of Chinese Medicine (Gansu Provincial Hospital), 730000 Lanzhou, Gansu, China

**Keywords:** acute coronary syndrome, metabolomics, metabolites

## Abstract

Acute coronary syndrome (ACS) is a severe type of coronary heart disease (CHD) with increasing prevalence and significant challenges for prevention and treatment. Metabolomics is an emerging technology with intrinsic dynamics and flexibility to better delineate the phenotypic and metabolic alterations in organisms at the time of altered pathological states. It provides new insights into the complex pathological mechanisms of cardiovascular disease and contributes to the early detection, monitoring and evaluation of ACS. In this review, we analyze and summarize the literature related to ACS metabolomics which has contributed to the diagnosis and prevention of ACS.

## 1. Introduction

Coronary heart disease (CHD) remains a major medical concern because of its high morbidity and mortality [1]. The morbidity and mortality of CHD have recently increased following a decline in Europe and the United States for some years [2]. Approximately 197 million CHD cases and 9.14 million CHD deaths were recorded worldwide in 2019 [3]. According to the China Cardiovascular Health and Disease Report 2021 [4], the prevalence of CHD in China continues to rise, with approximately 11.39 million CHD patients and a CHD mortality rate of 121.59/100,000 in urban and 130.14/100,000 in rural China in 2019.

Acute coronary syndrome (ACS) is a severe type of CHD, resulting in acute myocardial ischemia which may lead to necrosis. ACS as a multifactorial and multi-phenotypic disease is classified into unstable angina (UA), non-ST-segment elevation myocardial infarction (NSTEMI), and ST-segment elevation myocardial infarction (STEMI). It is the result of coronary artery occlusion which results in myocardial ischemic injury, which can be complicated by malignant arrhythmias, heart failure and even sudden death in severe cases.

## 2. Pathophysiology and Pathogenesis of Acute Coronary Syndrome

The main pathophysiological basis of ACS is acute thrombosis induced by unstable plaque rupture or endothelial erosion in coronary arteries, in which platelet activation plays an important role. The atherosclerotic plaque consists of cholesterol and macrophages which accumulate in the arterial wall over a long period of time. This process is insidious, and symptoms may occur over a prolonged period of time [5]. Elderly and diabetic patients often have atypical or “silent” clinical manifestations, which may lead to misdiagnosis. The diagnosis of ACS is currently based on clinical symptoms, electrocardiograms, myocardial plasma markers, and coronary angiography. However each of these tests has its limitations. In addition, some diseases present with symptoms very similar to ACS, such as Takotsubo syndrome (TTS), which often has an acute onset and has essentially the same electrocardiogram (ECG) presentation as ACS, with elevated myocardial enzymes such as troponin T(I) (TNT(I)) which are elevated several hours after onset and is often misdiagnosed as ACS. Therefore, it is essential to discover new biomarkers that can be identified early and quickly and have increased diagnostic and predictive value.

The heart is the most metabolically active organ in the body, and CHD is usually associated with energy imbalances resulting in metabolic dysfunction. Under normal conditions, cardiac metabolism is regulated by factors such as oxygen supply, substrate oxidation, hormonal and neurohumoral signals [6]. The main substrates for adenosine triphosphate (ATP) production are lipids, carbohydrates, lactate and glycogen, with lipids producing 60–90% of ATP in the heart *via* the oxidation of fatty acids [7]. Because of its widespread application in the myocardium and its important role in myocardial cell structure and cardiac function, alterations in lipid metabolism are closely associated with the development of many cardiovascular diseases [7]. A sudden reduction or interruption of coronary blood flow during ACS can lead to myocardial ischemia caused by an imbalance in myocardial metabolic supply and demand. During myocardial ischemia, a decrease in available oxygen and substrate performance alters the dynamic balance of cardiac metabolism, resulting in a loss of homeostasis. While some metabolic changes contribute to adaptive changes in the heart, most changes are maladaptive responses and can cause a range of cardiac abnormalities [7]. It has been shown that alterations in metabolites can also cause persistent disturbances in systemic metabolism [8]. Therefore, myocardial ischemia and metabolic alterations are key to the disease process of ACS. Early identification of these abnormal changes in metabolites and corresponding changes in metabolic patterns after ischemia may reveal important pathophysiological mechanisms of ACS and uncover potential targets for intervention.

## 3. Metabolomics Concepts and Development

Metabolomics is a new discipline that uses specific assays to assess the physiological and pathological states of an organism and identify the corresponding biometabolic markers and metabolic pathways of the corresponding physiological or pathological state, using metabolic substrates and products as the object of study. It is a technique for qualitative and quantitative analysis of low molecular weight metabolites in living biological cells over a specific period of time. The metabolome is intrinsically dynamic and flexible, so any perturbation in the level of metabolites is a true reflection of the phenotype and function of the developmental or pathological state of the organism. In a scientific statement, the American Heart Association [9] highlighted the potential impact of metabolomics on cardiovascular health and disease. Metabolomics has now emerged as an important tool for the detection of cardiovascular metabolites, and is well suited to assess various biological responses after ACS.

The metabolomic concept of determining diabetes by urine has been present since 1000 BC in China. Robinson *et al*.’s [7] pioneering laboratory work in the 1970s laid the preliminary foundation for metabolomic research. Oliver Fiehn [7] first used the term “metabolomics” in 1998 to denote changes in metabolite concentrations attributable to genetic abnormalities. A large number of analytical platforms with different sensitivities and specificities have been developed, however, due to the complex and dynamic nature of the metabolome, there is still no single platform that can simultaneously analyze all metabolites present in a biological sample. The commonly used analytical methods in metabolomics are nuclear magnetic resonance spectroscopy (NMR), gas chromatography with mass spectrometry (GC/MS) and liquid chromatography with mass spectrometry (LC/MS). These techniques can be combined to take advantage of each one. 13C-nuclear magnetic resonance spectroscopy (13C-NMR) and 31P-nuclear magnetic resonance spectroscopy (31P-NMR) are mainly applied in cellular energetics, for tracking changes in cellular myocardial metabolism [7], under normal and ischemic conditions [7]. NMR and MS use both non-targeted and targeted approaches for metabolomics studies.

## 4. Application of Metabolomics in ACS

Metabolomic investigations of ACS may help identify biomarkers for the early diagnosis of ACS. Detection of these clinical metabolomic markers will help to understand the metabolic pathways and potential mechanisms associated with the progression of ACS, and will be valuable for early detection and monitoring of the progression of the disease.

### 4.1 Sample Source for ACS Metabolomics

Depending on the pathophysiology of ACS disease, plasma or serum, is often used as a sample source for metabolomic studies, but it is unknown which source is better. Chacko *et al*. [10] in their study obtained coronary sinus and peripheral venous blood specimens to observe metabolic changes after acute myocardial ischemia. The same results were obtained from blood samples collected at both sites, confirming that these metabolic changes occur specifically in the myocardium. Urine metabolomics testing may also be an effective method to discover potential biomarkers of ACS and to explore the mechanisms of ACS [11]. Wang *et al*. [11] identified 9 metabolic pathways and 20 biomarkers of metabolites in urine samples, in which 11 metabolites were down-regulated in the urine of ACS patients, while the other 9 were up-regulated. Among these metabolic pathways, fatty acid metabolism, fatty acid β-oxidation metabolism, amino acid metabolism and tricarboxylic acid (TCA) cycle were closely associated with ACS (Table 1, Ref. [10, 11]).

**Table 1.** **Main clinical studies in Sample source of ACS metabolomics**.

No.	First author	Year	Specimen/technique	Sample num	Main findings
1	Sanoj Chacko [10]	2020	Plasma UPLC/MS	46 ACS	The same metabolic changes occur in both peripheral venous blood and coronary sinus blood
2	Yingfeng Wang [11]	2018	Urine UPLC/MS	36 ACS/30 Control group	Urine metabolomics testing may also be an effective method to discover metabolic changes of ACS

UPLC/MS, ultra-high performance liquid chromatography-tandem mass spectrometry; ACS, acute coronary syndrome.

### 4.2 Study on Lipid Metabolism in ACS

In addition to being the main structural component of cells, lipids play an important role in cell signaling molecules and energy supply. Fatty acid metabolism is the main source of energy for the heart [11]. Myocardial ischemia may cause a significant increase in free fatty acid concentrations. Genomic studies and large randomized controlled trials have confirmed the relationship between dysregulated lipid metabolism and the progression of coronary heart disease [7]. Wang *et al*. [11] showed that lipid-related pathways, including fatty acid metabolism and fatty acid β-oxidation, also play an important role in the course of ACS. In summary, elucidating the composition of lipids at the molecular level is necessary to characterize the molecular basis of ACS, to reveal lipid alterations during the development of ACS, and to find new biomarkers for the early diagnosis of ACS. Lee *et al*. [12] studied 365 lipids and found increased levels of saturated lysophosphatidylcholine (LPC) species in the high-density lipoprotein (HDL) fraction (16:0 and 18:0) in the ACS group, suggesting an intermediate link between LPC and ACS progression. Sutter *et al*. [13, 14] demonstrated the effect of altered HDL lipidome on the severity of ACS disease. Garcia *et al*. [15] using targeted lipidomics showed that the HDL2 subclass is more enriched in oxidized fatty acids in ACS patients than in non-ACS patients, which may increase the risk of platelet-dependent thrombosis. Wang *et al*. [11] found that the expression of the long-chain free fatty acid palmitic acid was upregulated in ACS patients. The elevated palmitic acid content in the urine samples of the ACS group reinforced the concept of altered energy metabolism and fatty acid β-oxidation in ACS patients.

Lysophosphatidylcholines (LysoPCs) are proinflammatory factors that contribute to atherosclerosis and are major components of oxidized low-density lipoprotein (LDL) [16]. LysoPCs induce inflammation, activate T lymphocytes and macrophages, alter vascular smooth muscle cells, activate intracellular protein kinase C (PKC), promote vascular cellular adhesion molecule-1 (VCAM-1) and intercellular cell adhesion molecule-1 (ICAM-1) expression, increase oxygen radical production, inhibit NO release from endothelial cells, and play an important role in the inflammatory response and remodeling of various cells in the vessel wall [17, 18]. Li *et al*. [19] found that increased levels of LysoPC were a marker for CHD in a model of myocardial ischemia. L-carnitine is an important cofactor for fatty acid B oxidation and plays an important role in promoting aerobic metabolism and scavenging toxic substances from energy metabolism [20, 21]. The content of L-carnitine and ATP in ischemic myocardium is significantly reduced, and free fatty acids are increased, leading to disturbances in myocardial cell energy metabolism [22]. Myocardial L-carnitine levels are significantly reduced, while blood L-carnitine levels are elevated, resulting from myocardial ischemia leading to partial release of L-carnitine into the blood or blocked transport of L-carnitine into cardiac myocytes [23]. Joanna *et al*. [24] using targeted analysis, demonstrated that ACS patients had the highest values for all 21 fatty acids on the day of onset of ACS, with values decreasing after 4 days and remaining at lower levels for 6 months.

Blood levels of trimethylamine oxide (TMAO) are an important marker for predicting ACS [25]. Lecithin, choline and carnitine can generate trimethylamine (TMA) through host gut microbes, which is subsequently released into the circulation to be oxidized by the liver to TMAO. This may promote the process of atherosclerosis by interfering with the reverse cholesterol transport. 3-dimethyl-1-butanol, on the other hand, can prevent ACS by interfering with TMA production in gut microbes and reducing circulating TMAO concentrations during ACS. Gut microbes are also thought to be key factors in regulating this process [25, 26]. Zhang *et al*. [27] performed a non-targeted analysis of metabolites in plasma in 2324 patients after coronary angiography, and quantitative analysis of N-acetylneuraminic acid using isotopic labeling, and also confirmed the role of N-acetylneuraminic acid in the pathophysiology of CHD (Table 2, Ref. [11, 12, 13, 14, 15, 19, 20, 24, 25, 27]).

**Table 2.** **Main clinical studies on lipid metabolism in ACS**.

No.	First author	Year	Specimen/technique	Sample num	Main findings
1	Yingfeng Wang [11]	2018	Urine	36 ACS/30 control group	Fatty acid metabolism and fatty acid β-oxidation play an important role in the course of ACS
			UPLC/MS		
2	Jae Hyun Lee [12]	2018	Plasma	365 lipids	The levels of saturated LPC species in the HDL fraction is increased in the ACS group
			UPLC-ESI-MS/MS		
3	Iryna Sutter [13]	2015	Blood	22 healthy subjects/23 stable CAD/22 ACS	The inverse association of HDL-plasmalogen levels with both stable and acute CAD may reflect direct anti-apoptotic effects of plasmalogens on ECs
			LC-MS/MS		
4	Fabiana Rached [14]	2015	Blood samples LC/MS	16 ACS/10 controls	HDL particle subpopulations display marked alterations in the early phase of STEMI
5	C Garcia [15]	2018	Plasma	30 ACS	The HDL2 subclass is more enriched in oxidized fatty acids in ACS, which may increase the risk of platelet-dependent thrombosis
			HPLC/MS		
6	Xinyuan Li [19]	2016	Tissues	8 *Apoe-/-* mice/5 Wild type mice atherosclerosis	Increased LysoPC was a marker of CHD development in a model of myocardial ischemia
			GC-MS		
7	Bridget M Stroup [20]	2018	Plasma/urin capillary GC/MS	25 adults with PKU/143 control	Bacterial degradation to TMAO
8	Joanna Teul [24]	2011	Blood	18 NSTEMI/6 control	The patient’s fatty acids in the ACS had a marked change
			GC-MS		
9	W.H. Wilson Tang [25]	2013	Plasma/urin	4007 CAD/40 controls	Blood levels of TMAO are an important marker for predicting ACS
			LC/MS		
10	Lei Zhang [27]	2018	Plasma	2324 CAD	N-acetylneuraminic acid play important role in the CHD process
			UPLC-Q/TOF-MS		

UPLC/MS, ultra-performance liquid chromatography-tandem mass spectrometry; ACS, acute coronary syndrome; UPLC-ESI-MS/MS, ultrahigh-performance liquid chromatography-electrospray ionization-tandem mass spectrometry; LC/MS, liquid chromatography with mass spectrometry; HPLC/MS, high-performance liquid-chromatographic with mass spectrometry; GC-MS, gas chromatography with mass spectrometry; UPLC-Q/TOF-MS, ultra performance liquid chromatography-quadrupole-time of flight-mass spectrometry; CAD, coronary artery disease; NSTEMI, non-ST segment elevation myocardial infarction; STEMI, ST-segment elevation myocardial infarction; PKU, phenylketonuria; HDL, high-density lipoprotein; LPC, lysophosphatidylcholine; ECs, endothelial cells; LysoPC, lysophosphatidyl cholines; CHD, coronary heart disease; TMAO, trimethylamine oxide.

### 4.3 Study of Amino Acid Metabolism in ACS 

The dependence of the normal heart on amino acids as a source of ATP is relatively small, but in patients with ACS, amino acids become an important source of energy and play an important role in ACS as precursors of energy metabolism [28]. Glutamate and glutamic acid can be phosphorylated at the substrate level to produce guanosine triphosphate. Yang *et al*. [29] suggested that branched-chain amino acids (BCAAs) predicted the risk for ACS. An increase in the concentration of BCAAs caused a significant increase in the risk of ACS development. Bernini *et al*. [30] also found that lower concentrations of threonine and creatinine anhydride reduced the risk of developing ACS. The essential amino acid tryptophan also plays an important role in human physiological and biochemical processes. Studies have shown that plasma choline levels are associated with an increased risk of cardiovascular disease [30, 31]. Yingfeng *et al*. [11] found that the upregulation of tryptophan in the urine of ACS patients was associated with a decrease in tryptophan catabolism, and that 5-hydroxytryptophan and 5-methoxytryptophan, the downstream metabolites of tryptophan, were also affected by changes in tryptophan content. Tryptophan can be converted to niacin, and its catabolic products, nicotinic acid and cucurbitacin, can affect choline metabolism. The study also demonstrated an upregulation of S-adenosyl-L-homocysteine expression. This suggests that the metabolic pathways of niacin and niacinamide were disrupted in the ACS patients, and that homocysteine is a potential marker in the progression of atherosclerosis (Table 3, Ref. [29, 30, 31]).

**Table 3.** **Main clinical studies on amino acid metabolism in ACS**.

No.	First author	Year	Specimen/technique	Sample num	Main findings
1	Ruiyue Yang [29]	2014	LC-MS/MS	472 CHD	An increase in the concentration of BCAAs caused a significant increase in the risk of ACS development.
2	Patrizia Bernini [30]	2011	Blood	864 healthy volunteers	Ower concentrations of threonine and creatinine anhydride reduced the risk of developing ACS.
			NMR		
3	Oliver Danne [31]	2007	Whole blood/plasma	217 suspected ACS	Plasma choline levels are associated with cardiovascular disease risk.
			HPLC/MS		

LC-MS, liquid chromatography with mass spectrometry; NMR, nuclear magnetic resonance; HPLC-MS, high performance liquid chromatography-tandem mass spectrometry; CHD, coronary heart disease; ACS, acute coronary syndrome; BCAAs, branched-chain amino acids.

### 4.4 Studies on Glucose and Other Metabolism in ACS

Myocardial ischemia and hypoxia occur in the acute coronary syndrome. Ischemia decreases aerobic oxidation of fatty acids, increases anaerobic respiration of glucose for ATP synthesis, and decreases aerobic metabolism of glucose while anaerobic glycolysis is enhanced [10]. Sabatine *et al*. [32] found that following an exercise stress test, there were significant changes in circulating levels of various metabolites such as lactate in ischemic myocardium. The pathway analysis suggested that the TCA cycle plays an important role in the process of myocardial ischemia. Li Jia *et al*. [33] found that succinate levels were abnormally elevated in the blood of ACS patients, and that ischemia and hypoxia can cause intracellular and extracellular succinate accumulation in the myocardium. Accumulation of intracellularly succinate inhibits pyruvate dehydrogenase (PDH) in a hypoxia-inducible factor -1α (HIF-1α)-dependent pathway, and extracellularly accumulated succinate activates its specific receptor G-protein-coupled receptor 91 (GPR91), which promotes downstream PKCδ activation and translocation to the mitochondria, impairing PDH activity, which affects the production of the TCA intermediate acetyl coenzyme A. Succinate-mediated blockade of glucose oxidation also exacerbates ischemia-reperfusion injury in cardiac myocytes. Another study [34] found that elevated levels of caffeine and paraxanthine caused coronary artery spasm leading to myocardial ischemia (Table 4, Ref. [32, 33, 34]).

**Table 4.** **Main clinical studies on glucose and other metabolism in ACS**.

No.	First author	Year	Specimen/technique	Sample num	Main findings
1	Marc S Sabatine [32]	2005	Plasma	18 inducible ischemia/18 controls	Significant changes in circulating levels of various metabolites and TCA cycle plays an important role in the process of myocardial ischemia.
			LC/MS		
2	Jia Li [33]	2017	Ventricular myocytes	ICR male mice	Succinate levels is closely related with ACS.
			Western blot/ELISA		
3	Xuejiao Yin [34]	2017	Plasma	20 ACS/20 non-ACS	Elevated levels of caffeine and paraxanthine caused coronary artery spasm.
			ICP-MS		

LC/MS, liquid chromatography with mass spectrometry; ICP-MS, inductively coupled plasma-mass spectrometry; ACS, acute coronary syndrome; TCA, tricarboxylic acid; ICR, randomly bred control.

### 4.5 Studies on Reperfusion Injury

Myocardial reperfusion injury caused by the sudden reintroduction of oxygen and nutrients during reperfusion in STEMI patients can exacerbate myocardial cell death. A sudden increase in available oxygen occurs after ischemia-reperfusion, resulting in a dramatic burst of mitochondrial reactive oxygen species that causes cellular dysfunction through modification of intracellular molecules. Ischemia-reperfusion also restores physiological pH, which inhibits the opening of the mitochondrial permeability transition space. Reperfusion can lead to intracellular calcium overload due to sarcoplasmic reticulum dysfunction, and increase endoplasmic reticulum stress which exerts a pro-inflammatory response [7] and activates pro-thrombotic pathways in ischemic tissues [7]. Even with rapid and successful reperfusion, the mortality rate after acute myocardial infarction (AMI) is still close to 10% [35]. Chouchani *et al*. [36] studied metabolomic assays in a mouse model of ischemia/reperfusion (I/R) injury and showed that succinate accumulation, an intermediate product of the TCA cycle during ischemia, and succinate oxidation during reperfusion, increased mitochondrial reactive oxygen species (ROS) and reperfusion injury. Kohlhauer *et al*. [37] using a targeted LC/MS approach to plasma metabolite analysis in STEMI patients treated with percutaneous coronary intervention (PCI), identified myocardial succinate accumulation as an early marker of I/R injury in humans. Li *et al*. [38] also showed that impaired BCAA catabolism inhibited glucose uptake and exacerbated I/R injury. Surendran *et al*. [7] found that pentadecanoic acid, 18:2 carnitine and, 18:2 lysophosphatidylcholine levels could determine the extent of I/R injury after PCI in STEMI patients. Jia *et al*. [33] found that ginsenosides could reduce succinate accumulation, restore PDH viability, and improve ischemia-reperfusion injury in cardiac myocytes. However, there are limited clinical treatment options for I/R injury [7]. Therefore, metabolomics will be used to find new methods to prevent and treat I/R injury (Table 5, Ref. [35, 36, 37, 38]).

**Table 5.** **Main clinical studies on reperfusion injury**.

No.	First author	Year	Specimen/technique	Sample num	Main findings
1	Muhammad Anas Kamleh [35]	2012	Urine	46 samples	Even with rapid and successful reperfusion, the mortality rate after AMI is still close to 10%.
			LC-MS		
2	Edward T Chouchani [36]	2014	Brain, kidney, liver and heart subjected	Murine	An intermediate product of the TCA cycle during ischemia, and succinate oxidation during reperfusion drove mitochondrial ROS accumulation and reperfusion injury.
			LC-MS		
3	Matthias Kohlhauer [37]	2018	Plasma	115 ASTEMI/26 controls	ASTEMI patients treated with PCI identified myocardial succinate accumulation as an early marker of I/R injury in humans.
			LC/MS		
4	Tao Li [38]	2017	Heart	Mouse	Impaired BCAA catabolism inhibited glucose uptake and exacerbated I/R injury.
			NMR		

LC-MS, liquid chromatography with mass spectrometry; AMI, acute myocardial infarction; ASTEMI, acute ST-segment elevation myocardial infarction; BCAA, branched-chain amino acids; PCI, percutaneous coronary intervention; TCA, tricarboxylic acid; NMR, nuclear magnetic resonance; I/R, ischemia/reperfusion.

### 4.6 Evidence on the Effects of Statin Therapy on Microbiota 

Statins are a class IA indication for secondary prevention in patients with CHD. Previous studies and clinical practice have demonstrated that the application of statin therapy is strongly associated with a reduced incidence of plaque rupture and improved prognosis for patients with ACS [39]. Hu *et al*. [39] found that patients on chronic statin therapy had fewer adverse events than those who had not received statins. This study used beta diversity analysis to find significant differences in microbial composition between the ACS and control groups. The BugBase database predicted that the ACS group was enriched in potentially pathogenic bacteria, while the ACS-statin group was relatively depleted, thus inferring that statins may be the driving force behind the shift of intestinal flora in ACS patients to a healthy population. This finding suggests that statins may reduce potentially pathogenic bacteria and increase probiotic bacteria such as bifidobacteria by modulating the intestinal flora of ACS patients. It also predicted that statins may be associated with fatty acid and isoprenoid pathways (Table 6, Ref. [39]).

**Table 6.** **Main clinical studies on the efficacy of statin therapy on microbiota**.

No.	First author	Year	Specimen/technique	Sample num	Main findings
1	Xiaomin Hu [39]	2021	Blood	30 control/67 ACS/36 ACS-statins	Statin treatment might benefit ACS patients by modulating the composition and function of the gut microbiome.
			HPLC-MS		

HPLC/MS, high-performance liquid-chromatographic with mass spectrometry; ACS, acute coronary syndrome.

### 4.7 Study of Coronary Artery Dilation in the Manifestation of ACS

The relationship between coronary atheromatous dilatation (CAE) and CHD is unclear. Coronary artery dilatation can lead to AMI and a poor prognosis. Swayze *et al*. [40] suggested that CAE may be a variant of CHD. Both diseases share similar risk factors, and both may also share a pathological process, one of the pathophysiological factors being the immune response to endothelial cell injury. Unfortunately, however, there are no studies on the metabolomics of CAE.

### 4.8 Studies on the Prognosis of ACS

Metabolomics has now made great progress in prognostic prediction [41]. Metabolomics has also been used to predict adverse cardiovascular events in patients with ACS. A study by Mehta *et al*. [42] using a non-targeted LC/MS approach, showed that six metabolic pathways, including the urea cycle, tyrosine, lysine, tryptophan, asparagine/aspartate and carnitine shuttle metabolism, were associated with increased mortality in CAD patients. Du *et al*. [43] performed LC/MS analysis of 26 amino acids in 138 patients with acute heart failure with an STEMI and found that elevated plasma BCAA levels at admission were associated with adverse cardiovascular events. Vignoli *et al*. [44] studied 2-year mortality in 978 patients with AMI using nuclear magnetic resonance spectroscopy methods and showed that elevated amino acid levels were strongly associated with mortality after an AMI. Chorell *et al*. [45] showed that the lysophospholipid ratio (lysophosphatidylcholine: lysophosphatidylethanolamine, LPC: LPE) was significantly associated with the risk for future cardiovascular adverse events in patients with STEMI and NSTEMI.

Plasma ceramides have been found to be predictive for the risk of ACS. Cheng *et al*. [46] studied lipids and 1-year clinical outcomes in 581 patients with ACS and stable CAD and showed that plasma ceramide (d18:1/16:0) was strongly associated with 1-year major adverse cardiac events (MACE) and plaque instability. Arterial and myocardial tissue ceramide levels were also associated with MACE in patients with an AMI. Carvalho *et al*. [47] further confirmed these results by performing a lipidomic analysis on paired tissue plasma samples in human and animal models. In a study with a mean follow-up of 3.1 years in 2023 patients with coronary angiography, they found a strong association between the dicarboxylation product acylcarnitine and the occurrence of death or MI, with an independent association with mortality [48]. A study on patients with coronary artery bypass grafts also suggested an association between short- to medium-chain dicarboxylated acylcarnitine and adverse cardiovascular events [49].

The value of metabolomics in predicting risk in patients with in-stent restenosis (ISR) has also been demonstrated. Cui *et al*. [50] studied the sensitivity and specificity of metabolites of phospholipids and sphingolipids for risk prediction in ISR patients, which were 91% and 90%, respectively. Cui *et al*. [51] also found that the metabolites arachidonic acid, sphingolipids and acylcarnitine predicted the recurrence of angina.

Jieying Luo *et al*. [52] studied the ultra-performance liquid chromatography/tandem mass spectrometry (UPLC/MS) platform approach to identify retinol metabolism as the best way to screen for early ventricular fibrillation (VF) after STEMI. 9-cis-retinoic acid (9cRA) was the most important biomarker to identify VF after STEMI (Table 7, Ref. [42, 43, 44, 45, 46, 47, 48, 50, 51, 52]).

**Table 7.** **Main clinical studies on the prognosis of ACS**.

No.	First author	Year	Specimen/technique	Sample num	Main findings
1	Anurag Mehta [42]	2020	Plasma	454 CHD	Six metabolic pathways (urea cycle, tyrosine, lysine, tryptophan, asparagine/aspartate and carnitine shuttle metabolism) were associated with mortality in CAD patients.
			LC/MS		
2	Xiaoyu Du [43]	2018	Plasma	138 STEMI and AHF	Increased plasma BCAA levels are associated with long-term adverse cardiovascular events.
			MS		
3	Alessia Vignoli [44]	2019	Serum	978 AMI	Elevated amino acid levels were strongly associated with mortality after AMI.
			1H-NMR		
4	Elin Chorell [45]	2021	Plasma	50 STEMI+50 NSTEMI/100 controls	Lysophospholipid ratio was significantly associated with future cardiovascular risk in patients with STEMI and NSTEMI.
			MS		
5	Jin M Cheng [46]	2015	Plasma	581 CHD	Plasma ceramide was strongly associated with MACE and plaque vulnerability.
			IVUS-VH/NIRS		
6	Leonardo P de Carvalho [47]	2018	Plasma	337 AMI	A plasma signature of ceramides and dihydroceramide predictive of major adverse cardiovascular events.
			HILIC/MS/MS		
7	Svati H Shah [48]	2012	Plasma	2023 patients undergoing CAG	A strong association between the dicarboxylation product acylcarnitine and the occurrence of death or MI.
			MS		
8	Song Cui [50]	2017	Plasma	400 patients undergoing PCI	Phospholipid and sphingolipid metabolites have high predictive value for ISR.
			HPLC		
9	Song Cui [51]	2021	Plasma	1655 CHD	The metabolites arachidonic acid, sphingolipids and acylcarnitine predicted angina recurrence.
			LC-MS/MS		
10	Jieying Luo [52]	2022	Plasma	42 STEMI	9cRA was the most important predictive biomarker of VF.
			UPLC/MS		

LC/MS, liquid chromatography with mass spectrometry; CHD, coronary atherosclerotic heart disease; CAD, coronary artery disease; STEMI, ST-segment elevation myocardial infarction; AHF, acute heart failure; BCAA, branched-chain amino acids; NMR, nuclear magnetic resonance; AMI, acute myocardial infarction; NSTEMI, non-ST segment elevation myocardial infarction; IVUS-VH/NIRS, intravascular ultrasound-virtual histology with near-infrared spectroscopy; MACE, major adverse cardiac events; HILIC, hydrophilic interaction chromatography; CAG, coronary angiography; HPLC, high-performance liquid-chromatographic; PCI, percutaneous coronary intervention; ISR, in-stent restenosis; UPLC/MS, ultra-performance liquid chromatography/tandem mass spectrometry; VF, ventricular fibrillation; ACS, acute coronary syndrome; MI, myocardial infarction.

## 5. Current Limitations and Dilemmas of Metabolomics

Despite the great potential of metabolomics studies in discovering biomarkers and pathogenesis of ACS, few metabolomics findings are currently translated specifically into disease diagnosis and risk prediction due to the diversity and reliability of marker/metabolite clusters. In addition, most of the current metabolomics studies in ACS are preliminary results of small-scale studies.

## 6. Conclusions

Metabolomics is currently at an early stage of development. Despite some limitations, it is inherently more sensitive, faster, and more accurate, and it is believed that it will play an important role in the early diagnosis, treatment, and prognostic assessment of ACS in the future as advancements are made with this technology.
